# The Rotary Cell Culture System increases NTRK3 expression and promotes neuronal differentiation and migratory ability of neural stem cells cultured on collagen sponge

**DOI:** 10.1186/s13287-021-02381-y

**Published:** 2021-05-21

**Authors:** Yi Cui, Yanyun Yin, Yunlong Zou, Yannan Zhao, Jin Han, Bai Xu, Bing Chen, Zhifeng Xiao, Hongwei Song, Ya Shi, Weiwei Xue, Xu Ma, Jianwu Dai

**Affiliations:** 1 Reproductive and Genetic Center of National Research Institute for Family Planning, Beijing, 100081 China; 2 Key Laboratory of Molecular Developmental Biology, Institute of Genetics and Developmental Biology, Chinese Academy of Sciences, 3 Nanyitiao, Zhongguancun, Beijing, 100190 China; 3 Orthopaedics Surgery Department, China-Japan Union Hospital of Jilin University, No. 126, Xiantai Street, Changchun, 130033 Jilin Province China; 4 EHBIO gene technology, No. 46, Jiugulou Street, Beijing, 100100 China

**Keywords:** RCCS, NSCs, NTRK3, let-7i-5p, Collagen sponge scaffold

## Abstract

**Background:**

Recently, neural stem cell (NSC) therapy has shown promise for the treatment of many neurological diseases. Enhancing the quality of implanted cells and improving therapeutic efficacy are currently research hotspots. It has been reported that collagen sponge material provided sufficient room for cell growth in all directions and promoted the absorption of nutrients and removal of wastes. And also, the Rotary Cell Culture System (RCCS), which mimics the microgravity environment, can be used to culture cells for tissue engineering.

**Materials and methods:**

We performed the mRNA and miRNA sequencing to elucidate the regulatory mechanism of NSCs cultured on the collagen sponge in the RCCS system. The luciferase assay and Western blot revealed a direct regulatory role between let-7i-5p and neurotrophic receptor tyrosine kinase 3 (NTRK3; also called TrkC). And then, the neural differentiation markers Tuj1 and Map2 were detected by immunofluorescence staining. In the meantime, the migratory ability of NSCs was detected both in vitro and in spinal cord injury animals.

**Results:**

In this study, we demonstrated that the expression of NTRK3 was elevated in NSCs cultured on collagen sponge in the RCCS system. Furthermore, increased NTRK3 expression was regulated by the downregulation of let-7i-5p. Compared to traditionally cultured NSCs, the NSCs cultured on collagen sponge in the RCCS system exhibited better neuronal differentiation and migratory ability, especially in the presence of NT-3.

**Conclusions:**

As the biological properties and quality of transplanted cells are critical for therapeutic success, the RCCS system combined with the collagen sponge culture system shows promise for applications in clinical practice in the future.

## Background

Recently, the transplantation of neural stem cells (NSCs) for the treatment of nervous system diseases has gained attention in neuroscience research [1,3]. NSCs are capable of self-renewal and differentiation and thus hold great promise for nervous system regeneration. As an appropriate cell source for transplantation, NSCs have always been considered suitable for the treatment of many neurodegenerative diseases and traumatic injuries, such as Alzheimers disease, Parkinsons disease, traumatic brain injury, and spinal cord injury [4,8]. However, the major concern regarding cell transplantation is that many implanted NSCs differentiate into glial cells but not functional neuronal cells. Therefore, discovering methods to overcome these obstacles is crucial.

Our previous studies have demonstrated that the conditions in outer space promote neuronal differentiation of NSCs [9]. As microgravity is one of the key factors of outer space [10, 11], this present study investigated the influence of simulated microgravity on three-dimensional (3D) cultured NSCs with a dynamic 3D Rotary Cell Culture System (RCCS). The microgravity environment within the RCCS bioreactor is provided by using a random positioning machine that produces low fluid shear force. It is based on a continuous rotation of the culture chamber around its horizontal axis, avoiding drops to the bottom of the chamber [12,14]. Here, we selected the collagen sponge scaffold as the cell carrier since it provides a good microenvironment for cell growth and differentiation. The porous network structure of the collagen sponge scaffold helps transport cellular nutritive substances and metabolic waste [15,17]. Moreover, regarding animal experiments, a single injection of NSCs may be washed away from the transplantation site by blood, tissue fluid, or cerebrospinal fluid. Conversely, when cells are anchored to the scaffold, NSCs can be easily placed at the injury site.

In this study, we detected that neurotrophic receptor tyrosine kinase 3 (NTRK3) expression was elevated in 3D cultured NSCs in a microgravity environment. The therapeutic potential of neurotrophin-3 (NT-3) has been extensively studied in central nervous system (CNS) injury models. The protective effect of NT-3 was mainly mediated via activation of NTRK3, which may bind specifically and with high affinity to NT-3. Exogenous application of NT-3 has the potential to create a growth-permissive environment for the treatment of neurological disorders [18,20]. Therefore, the expression level of NTRK3 was related to the role and effect of NT-3. The elevated expression of NTRK3 in 3D cultured NSCs in a RCCS bioreactor would improve the positive role of NT-3 on NSCs.

Our study promised to offer an insight into the molecular mechanism of how the effect of the RCCS system on the biological characters of NSCs compared to traditional static culture systems, as well as to know whether elevated NTRK3 expression in microgravity culture conditions may facilitate neuronal differentiation and the migratory ability of NSCs. And then, the 3-D cultured NSCs in the RCCS system may provide an attractive solution for therapeutic cell therapy which will improve the therapeutic effects of transplanted NSCs.

## Methods

### NSC isolation and culture

In accordance with a previous report [21], brain hemispheres were dissected and separated by the trypsin enzyme-digesting technique in newborn rats (within 12 h after birth). All animal procedures were performed in accordance with the Chinese Ministry of Public Health Guide for the care and use of laboratory animals. Single cells were placed in a growth medium with B27 supplement (Invitrogen, Carlsbad, CA, www.invitrogen.com), epidermal growth factor (20 ng/ml, Sigma-Aldrich), and basic fibroblast growth factor (20 ng/ml, Sigma-Aldrich). After 7 days, the NSCs proliferated and formed neurospheres and were harvested and digested by TrypLE Express Enzyme. For 2D cultures, the cells were seeded onto poly-D-lysine-coated dishes. For 3D cultures, 1 10^6^ NSCs were seeded onto each collagen sponge scaffold. The next day, the adhesion medium was replaced with a traditional differentiation medium with B27 supplement (Invitrogen, Carlsbad, CA, www.invitrogen.com) and without growth factors. The 3D cultured cells were divided into four groups: static (cultured in traditional differentiation medium), RCCS (cultured in traditional differentiation medium), static+NT-3 (cultured in differentiation medium with NT-3 (50 ng/ml), Peprotech Inc., CA, USA), and RCCS+NT-3 (cultured in differentiation medium with NT-3 (50 ng/ml), Peprotech Inc., CA, USA). The 3D cultured cells were harvested for subsequent analysis after 12 days in culture.

### Animal experiments

Sprague-Dawley rats (200250 g) were purchased by Charles River Laboratories (Beijing). All animal experimental procedures were performed in accordance with the Chinese Ministry of Public Health guide for the care and use of laboratory animals. The SD rats were anesthetized with intraperitoneal injections of ketamine (80 mg/kg) and xylazine (10 mg/kg). Following the removal of the vertebral lamina, the T10 spinal cord was exposed and cut off completely using microsurgery scissors. A total length of 1 mm of the spinal cord was removed, and the injury defect was bridged by a collagen scaffold with/or without cells. The cells that were implanted into the animal models were transfected with the green fluorescent protein (GFP) recombinant adenovirus vector. Bladder emptying and antibiotic treatment were performed routinely after surgery. The rats were sacrificed 2 weeks after surgery for immunofluorescent staining.

### Preparation of collagen sponge scaffolds

As previously reported [15], the collagen sponge scaffold was processed from bovine cancellous bone, which had been demonstrated to be an ideal scaffold for culturing cells. The collagen sponge scaffold was 1 mm thick and 5 mm in diameter. The surface appearance of the collagen sponge scaffold was observed by a scanning electron microscope (SEM) (S-3000N; Hitachi, Tokyo, Japan).

### SEM analysis

To assess cell morphology, the 3D cultured cells were fixed in 2% glutaraldehyde at 4 C overnight and then dehydrated with the different concentration ethanol. Following underwent conventional approaches and critical point drying, samples were sprayed with gold and then evaluated under SEM (S-3000N; Hitachi).

### Fluorescein diacetate NSC staining

Fluorescein diacetate (FDA) can be used to assess the cell viability of numerous cell types, as it generates observable differences in the fluorescence produced by live versus dead cells [22]. The collagen scaffold with implanted cells was incubated with 100 g/ml FDA for 1 min in the dark and then rinsed with phosphate-buffered saline. Samples were observed using the Zeiss 200 inverted fluorescent microscope (Carl Zeiss, Jena, Germany).

### The miRNA-mRNA regulatory network

RNA sequencing was performed by Novogene company (Beijing, China). Differentially expressed mRNAs were screened by a cut-off fold-change of 1.5 and a P value of < 0.01 in the Students t-test. The predicted interplay network between these differentially expressed mRNAs and miRNAs involved in regulating them was constructed using Cytoscape (version 3.0.1). The target gene of miRNA was predicted by TargetScan (release 7.0).

### Luciferase reporter assays

We performed luciferase assays according to the manufacturers instructions. To construct the let-7i reporter plasmid, the wild and mutant fragment of the NTRK3 3-UTR (160 bp) gene containing the putative let-7i binding site was cloned into the pmirGLO Dual-Luciferase miRNA Target Expression Vector (Promega, Madison, WI, USA). The inserted gene segments were synthesized by Generay Biotechnology (Shanghai, China). Hela cells were seeded onto 96-well plates and transfected using Lipofectamine 3000 reagent (Invitrogen, Carlsbad, CA, USA). Firefly luciferase vector (0.1 g) and let-7i-5p mimics (2.5 nM) or let-7i-5p inhibitors (2.5 nM) were added to each well. After 48 h, firefly and Renilla luciferase activities were measured by dual-luciferase assays (Promega, Madison, WI, USA). All luciferase data were calculated as a normalized ratio of luciferase/Renilla.

### Real-time PCR

Total RNA was isolated with TRIzol Reagent (Invitrogen) according to the manufacturers instructions. The NTRK3 primers used were 5-GTGAATGTGACGAGCGAAGA-3 and 5-GGAGGGTAGTAGACAGTGAGAG-3. RT-PCR was performed with a CFX96 Real-Time PCR Detection System (Bio-Rad Laboratories Co., Hercules, CA, USA). Relative mRNA levels were calculated and standardized with the GAPDH internal control using the 2^CT^ method. The extraction of miRNAs was performed using the miRVana extraction kit (Ambion, Austin, TX, USA). The reverse transcription process was performed using the Taqman miRNA Reverse Transcription Kit and specific let-7i-5p primers (ThermoFisher, CA, USA). All results were normalized to U6 levels and calculated using the 2^CT^ method (Applied Biosystems, Inc. Foster City, CA).

### miRNA mimics, inhibitors, overexpression plasmid, and small interfering RNA transfection

The expression of let-7i was regulated using chemically synthesized mimics or inhibitors, which were designed and purchased from Ribobio (Ribobio Co., Ltd., Guangdong, China). The sequences of small interfering RNA (siRNA) duplex for NTRK3 were also purchased from Ribobio company (Guangdong, China). Cells were divided into five groups transfected with miRNA mimics, miRNA inhibitors, RNAi, and overexpression plasmid separately. NTRK3 siRNA and overexpression plasmid were transfected using Lipofectamine 3000 following the manufacturers instructions.

### Western blot analysis

Western blot analysis was used to demonstrate the modulation of let-7i-5p on NTRK3. The transfected cells were lysed and homogenized in RIPA buffer. Approximately 20 g protein was loaded onto 10% polyacrylamide gels and separated on the Bio-Rad mini gel system (Hercules, CA). Primary antibodies of NTRK3 (gtx83316, GeneTex) and GAPDH (gtx100118, GeneTex) were incubated overnight at 4 C. The HRP-labeled secondary antibody (1:5000; PIERCE, Rockford, USA) was incubated for 2 h at room temperature. Bands were visualized with colorimetry using a chemiluminescent imaging analyzer (Tanon 5200, Tanon Science & Technology Co., Ltd., Shanghai, China).

### Immunofluorescence staining

To evaluate NSC differentiation, neuronal differentiation-related markers were detected by immunofluorescence staining. Cells or frozen sections of Sprague-Dawley (SD) rat spinal cord tissues were fixed with 4% paraformaldehyde and then pretreated with 0.8% Triton X-100 to increase the permeability of the cell membrane. The primary antibodies anti-Tuj1, anti-MAP2, and anti-GFAP (1:1000, Millipore, Temecula, CA, USA) were applied overnight at 4 C. This was followed by three washes with phosphate-buffered saline and then incubation with secondary antibodies at room temperature for 2h. An inverted fluorescent microscope (Zeiss 200, Carl Zeiss, Jena, Germany) was used to capture the fluorescent images of 2D cultured cells. The fluorescent images of 3D cultured cells and frozen sections were observed by a scanning laser confocal fluorescence microscope (Leica TCS SP5, Leica Microsystems, Inc., Germany).

### Cell migration assay

The cell migration assay for NSCs was performed in a 24-well plate Boyden chamber (Corning-3422, Corning Costar Corp, Cambridge, MA, USA). After culturing for 14 days, the cells were harvested from the collagen scaffold by collagenase type I (Invitrogen, Carlsbad, CA). The single cell suspension (1 10^4^ cells) was placed in the upper chamber that had been coated with poly-D-lysine. The proliferation medium in the lower chamber contained 0.5 g/ml SDF-1 (R&D systems, MA, USA). Following incubation for 24 h at 37 C, the cells on the upper surface of the membrane were scrubbed by a cotton swab. The bottom side of the membrane was fixed with 4% paraformaldehyde and stained with hematoxylin and eosin. The total number of migrated cells was counted using a light microscope. The stained cells were calculated from three randomly chosen fields at a magnification of 20.

### Statistical analyses

All results were statistically analyzed and presented as mean SD from at least three independent experiments. Students *t*-test and factorial analysis of variance (ANOVA) were analyzed with SPSS (SPSS version 13.0; SPSS Inc., Chicago, IL, USA). Other analyses were performed using Prism 3.0 (Graphpad Software Inc., San Diego, CA, USA).

## Results

### The morphology and good cytocompatibility of the collagen sponge scaffold

**Fig. 1:**
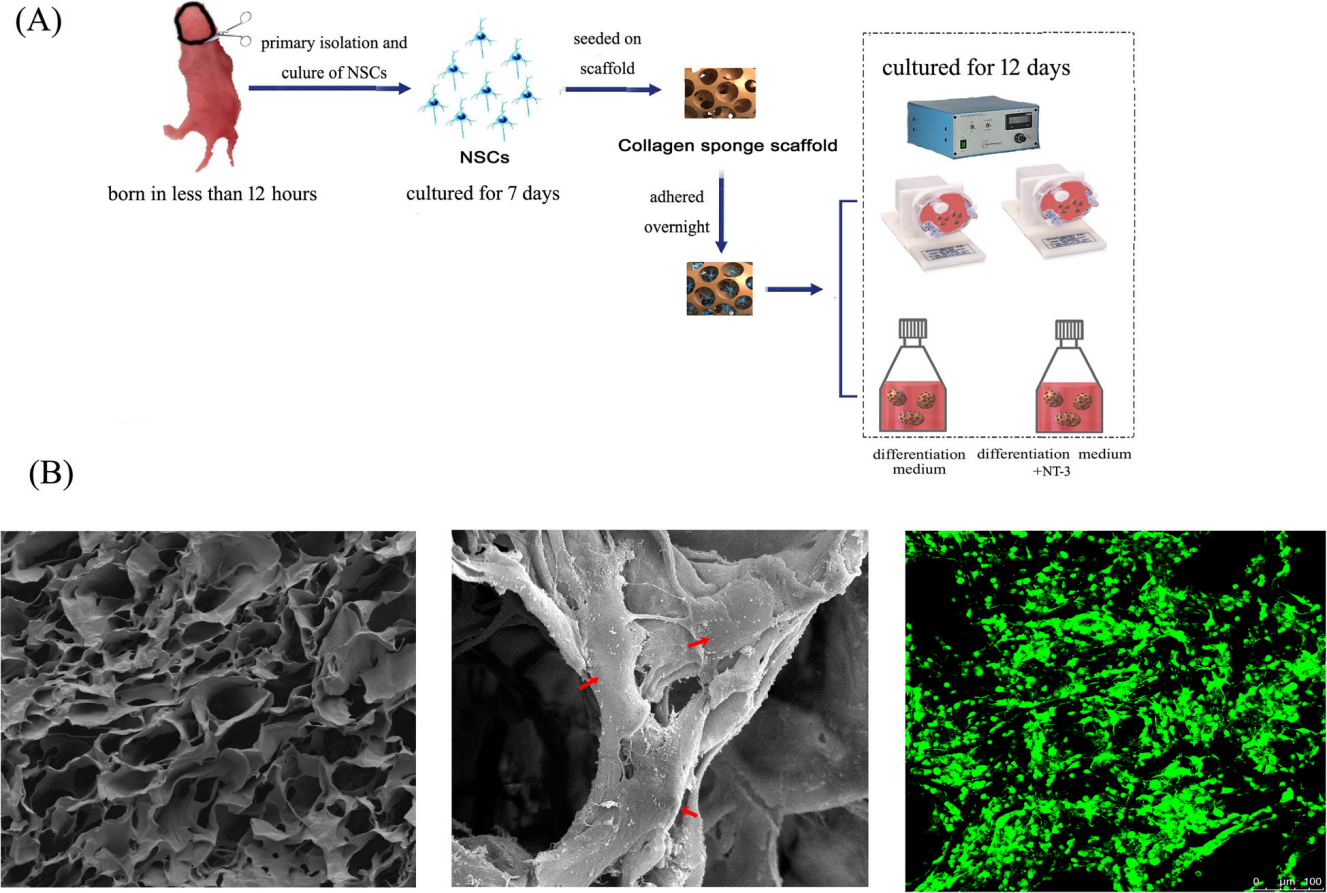
**a** A flowchart of the experiment. **b** Scanning electron microscopy images reveal the morphology of the collagen sponge scaffold (left) and 3D cultured neural stem cells (NSCs) (middle). The red arrow indicates a cell that attached to the scaffold. A representative cell image of fluorescein diacetate staining of the 3D cultured NSCs (right) under the traditional static culture environment

SEM revealed that the collagen sponge scaffold possesses a rich internal porous structure and good porosity (Fig.1a). The porous structure contributes to the transport of oxygen, nutrients, and waste. The average pore diameter was 40.69 m with good porosity. The 3D structure allows the cells to stretch out in all directions. Good NSC adhesion and growth were observed on the surface and in the pores of the collagen sponge scaffold (Fig.1b). FDA staining also revealed that the cells attached and spread well on the collagen sponge scaffold (Fig.1c). These results indicate that the collagen sponge scaffold represents an excellent alternative to 2D culturing of NSCs.

### The effect of the RCCS system on the cellular properties of NSCs

**Fig. 2:**
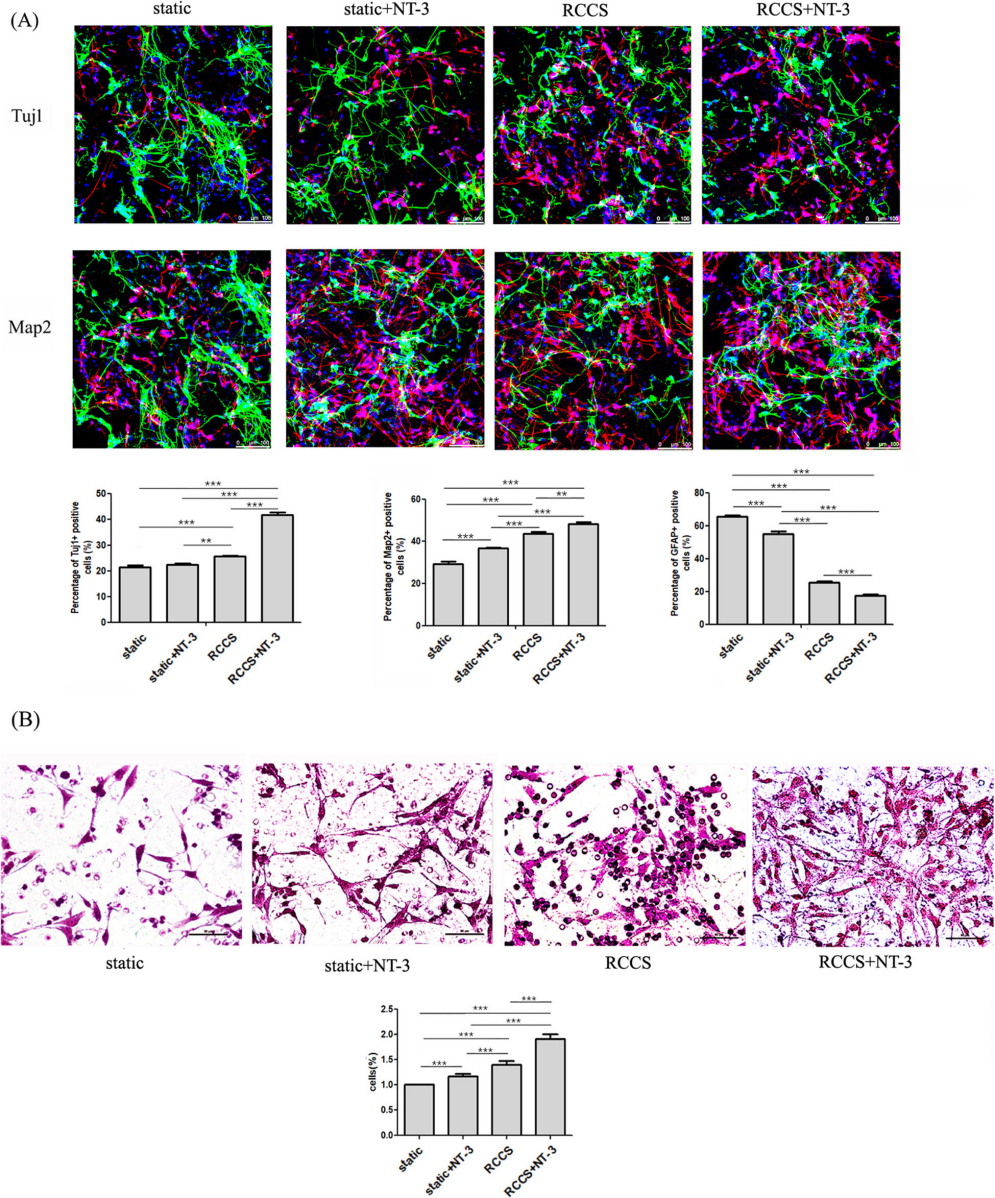
The differentiation and migratory ability of 3D cultured NSCs in all groups detected by immunofluorescence staining (**a**) and trans-well migration array (**b**)

Previous studies have reported that microgravity can alter cellular properties. Here, we evaluated the effect of simulated microgravity on the cellular properties of NSCs. Immunofluorescence staining (Fig.2a) and the migration assay (Fig.2b) indicated, compared to the control group, increased neuronal differentiation and migration and decreased GFAP expression of 3D cultured NSCs in the RCCS system. Immunofluorescence staining revealed that the NSCs cultured in the RCCS bioreactor exhibited enhanced neuronal differentiation and suppression of astrocytic differentiation in the NT3-containing differentiation medium.

**Fig. 3:**
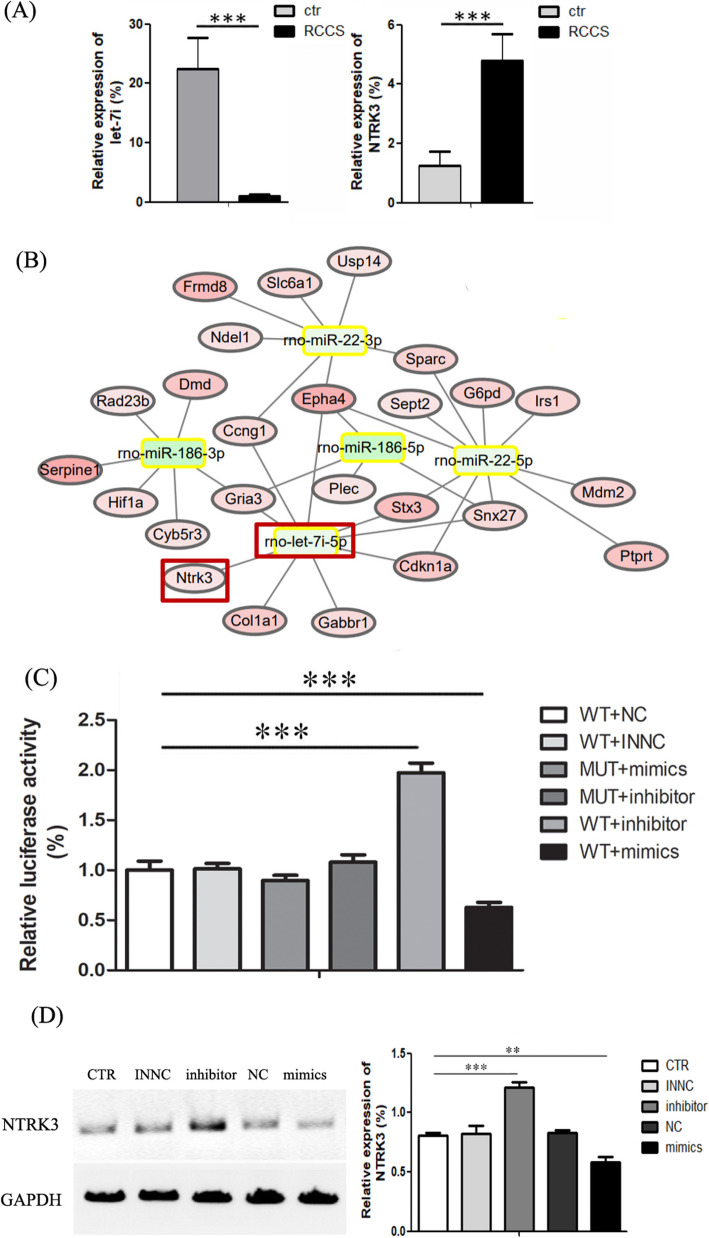
The interaction between let-7i-5p and NTRK3. **a** Bioinformatics analysis predicted a regulatory role of let-7i-5p on NTRK3. **b** Real-time PCR indicates let-7i-5p and NTRK3 expression in the RCCS and control groups. **c** The luciferase reporter array indicated an interaction between 3-UTR of NTRK3 and the seed sequence of let-7i-5p. **d** Western blot indicated that NTRK3 protein levels are affected by let-7i-5p mimics and inhibitors

Both real-time PCR and RNA sequencing showed increased NTRK3 expression in the RCCS group compared to the control group (Fig.3b). Thus, we further evaluated the effect of simulated microgravity on NSCs in the presence of NT-3. There is a large amount of evidence that NTRK3 is widely expressed in the brain and lumbar spinal cord and has a high affinity for NT-3 [23,25]. In the RCCS system, neuronal differentiation and migratory ability were increased, whereas the differentiation ability of astrocytes was decreased in NT-3-containing versus traditional medium. Taken together, these results indicate that the RCCS system contributes to the neuronal differentiation and migration of NSCs, especially in the differentiation medium containing NT-3.

### The direct regulatory role between let-7i-5p and NTRK3

The analysis of mRNA sequencing data identified some differentially expressed mRNAs in NSCs between the traditional and RCCS cell culture systems. This altered mRNA expression may elucidate the mechanisms underlying the effects of the RCCS system on 3D cultured NSCs. Using the miRNA target gene pairs predicted by TargetScan, we constructed the regulatory network between differentiated expressed mRNAs and miRNAs predicted to be involved in regulating them. Among the regulatory network, the regulatory relationship between NTRK3 and let-7i-5p caught our attention. The bioinformatics analysis indicated that the upregulation of NTRK3 may be regulated by the downregulation of let-7i-5p (Fig.3a). To prove the accuracy of the RNA sequence data, we performed quantitative real-time PCR (Q-PCR). Consistent with the RNA sequencing data, Q-PCR showed that the relative expression of NTRK3 increased by fourfold in the RCCS group, whereas the relative expression of let-7i-5p decreased by nearly 20% (Fig.3b, c).

Considering that the bioinformatics analysis indicated that the 3UTR of the NTRK3 gene contained the let-7i-5p binding sites, we performed the luciferase report assay to test the interaction between them. Luc activity was the lowest in the wild plasmid combined with the let-7i-5p mimics for Rest UTRs in HeLa cells, whereas significantly upregulated in the wild plasmid combined with let-7i-5p inhibitors (Fig.3b). When the binding site in the wild plasmid was mutated, no significant alteration was detected when combined with let-7i-5p mimics. These results suggest that the NTRK3 binding site is required for miRNA binding and activity. The Western blot analysis further validated the regulatory role between let-7i-5p and NTRK3 in NSCs. Compared to the control group, the NTRK3 protein level was decreased in the let-7i-5p inhibitor group and increased in the let-7i-5p mimic group (Fig.3d).

### Let-7i-5p regulates NSC activity by targeting NTRK3 in NT3-containing differentiation medium

**Fig. 4:**
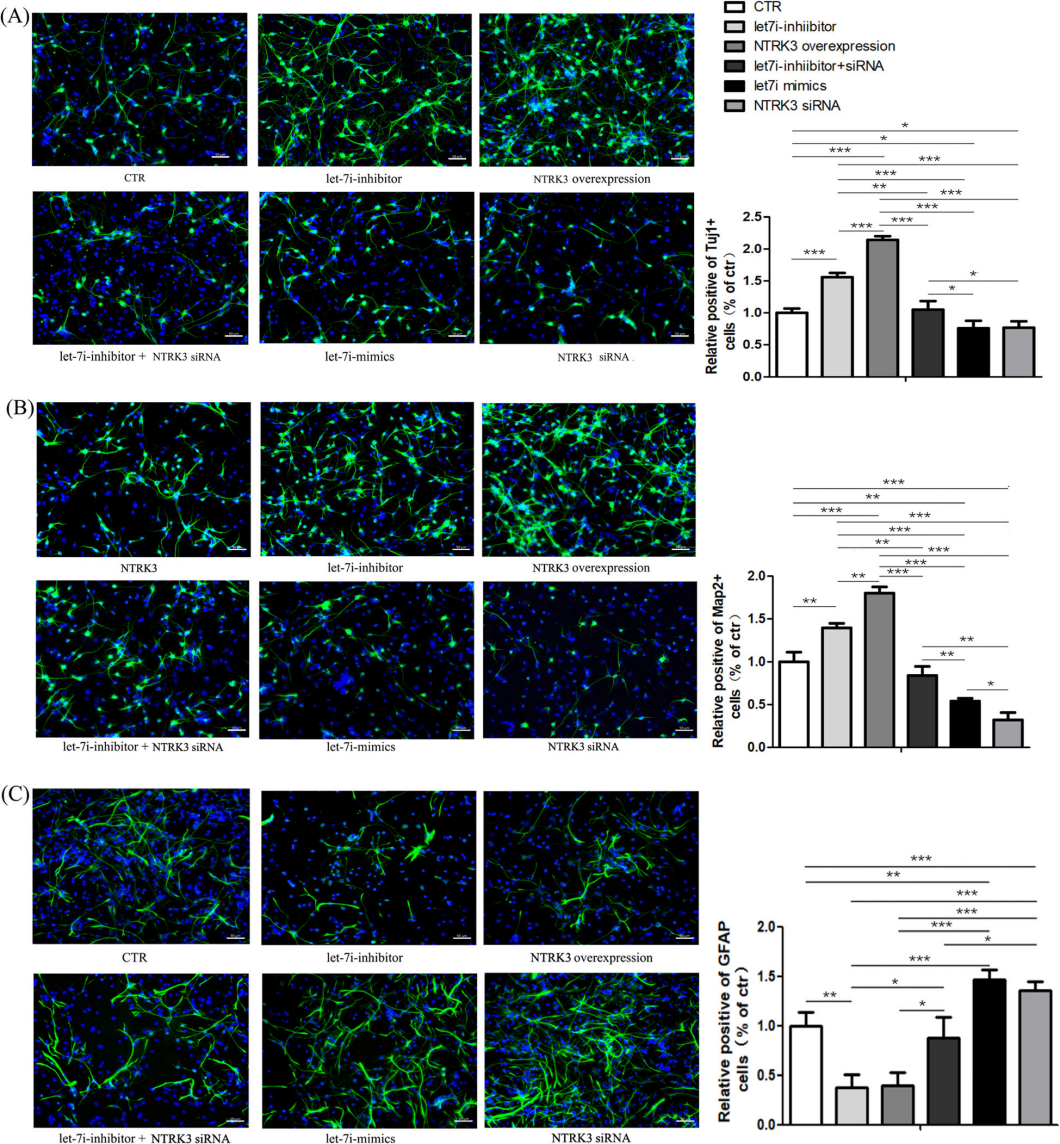
Immunofluorescence staining indicated an effect of let-7i-5p and NTRK3 on NSC differentiation when the differentiation medium contained NT3; **a** neuronal class III -tubulin (Tuj1), **b** microtubule-associated protein 2 (Map2), **c** glial fibrillary acidic protein (GFAP)

**Fig. 5:**
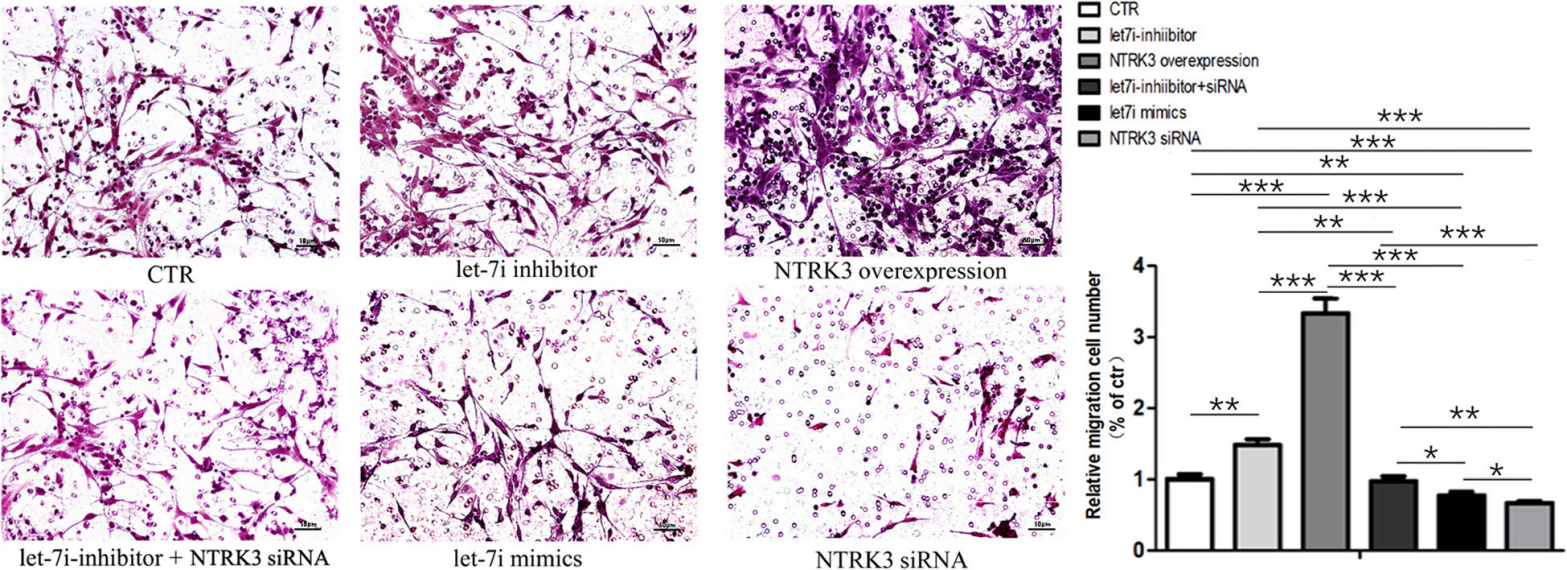
The trans-well migration assay indicated an effect of let-7i-5p and NTRK3 on NSC migration in the presence of NT3

To further validate the regulatory roles of let-7i-5p and NTRK3, we performed immunofluorescence staining and trans-well migration assay to compare the differentiation (Fig.4) and migratory (Fig.5) ability of NSCs between six groups: ctr, let-7i-5p mimics, let-7i-5p inhibitors, NTRK3 siRNA, NTRK3 overexpression, and let-7i-5p inhibitor+NTRK3 siRNA. The NT3-containing medium was used in all groups. Consistent with NSCs transfected with let-7i-inhibitor, the neuronal differentiation and migratory ability of NSCs transfected with NTRK3 overexpression plasmid was enhanced, accompanied with a decreased ability for astrocyte differentiation. Meanwhile, neuronal differentiation and migratory ability were poor in the let-7i-5p-mimics and NTRK3 siRNA groups. Additionally, the positive effect of the let-7i-5p-inhibitor on neuronal differentiation and migration can be reversed when combined with NTRK3 siRNA. When NTRK3 was knocked down by siRNA, the boost from the let-7i-5p-inhibitor was undermined. As such, these results suggest that NTRK3 is a target gene of let-7i-5p and plays a certain role in regulating neuronal differentiation and migration, especially when exposed to exogenous NT3. Overexpression or knockdown of let-7i-5p in NSCs further indicated that let-7i plays an inhibitory role on the expression of its target gene NTRK3. Taken together, we have demonstrated for the first time that microRNA let-7i-5p is a negative regulator of NTRK3 in NSCs.

### The migratory ability of implanted NSCs in animal models of spinal cord injury (SCI)

**Fig. 6:**
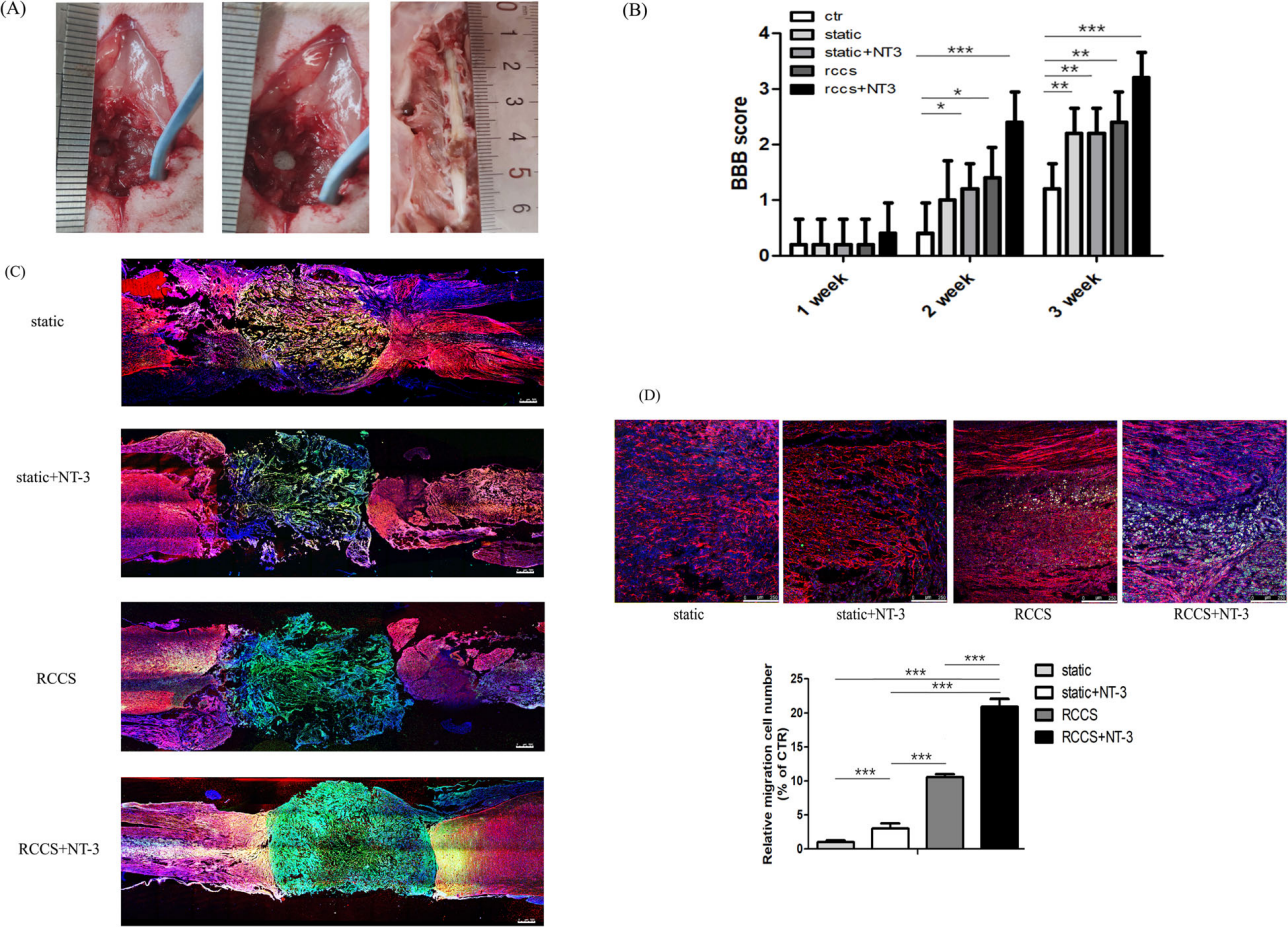
The transplantation of NSCs in rats with spinal cord injury. **a** Spinal cord injuries (with lengths of 3 mm) were inflicted in rats (left). Functional collagen sponge scaffolds with or without cells were precisely placed into the injury site (middle). Samples of the spinal cord tissue following perfused 4% paraformaldehyde fixation (right). **b** The BBB scores of various groups. Locomotor function recovery in five groups at 1, 2 and 3 weeks after spinal cord injury. **c** Images of immunofluorescence staining of spinal injury sections at 2 weeks from different groups of SCI rats, stained with antibodies against GFAP (red) and with DAPI (blue). The green signal represents the GFP-labelled transplanted NSCs. Scale bars: 500 m. **d** Enlarged images of the migrated GFP^+^ cells in the RCCS and RCCS+NT-3 groups. Quantification of the number of migrated cells showing that the NSCs in RCCS+NT-3 group possess the best migratory ability in SCI animals. Scale bars: 250 m. Data represent meanS.D. from three independent experiments. ***p < 0.001

To further assess the migratory ability of 3D cultured NSCs in different groups, we then implanted collagen sponge scaffold with cells at the injury site in rats with spinal cord injury (Fig.6a). Immunofluorescent staining indicated that the implanted cells still remained within the implanted injury site in both the static and static+NT-3 groups 2 weeks after implantation (Fig.6c). Conversely, a small fraction of cells in the RCCS and RCCS+NT-3 groups migrated from the implanted lesion site to the injury site. Compared with the RCCS group, the RCCS+NT-3 group exhibited more NSC migration and with longer distances (Fig.6d). Consistent with the cell experiments, the in vivo animal tests further demonstrated that 3D cultured NSCs in the RCCS environment possess better migratory ability than that of other groups, especially in the presence of NT-3. The migratory ability of NSCs in the RCCS+NT3 group was enhanced compared to that of other groups at the injury site, indicating that NT3 may improve the therapeutic effects of transplanted NSCs.

## Discussion

Due to the lack of regeneration in the CNS, spontaneous repair is restricted after injury or degeneration. The transplantation of NSCs has a promising future both for stem cell therapy and scientific research. Currently, cell therapy is a hotspot in neuroscience research. Multiple preclinical trials have convincingly demonstrated that NSC transplantation promotes a positive outcome (i.e., neurogenesis) in rodent models, indicating a novel approach for the treatment of various CNS diseases. Nevertheless, the promising application of NSC transplantation still remains elusive, since many grafted cells differentiate into glial cells, which form scars instead of repairing the damaged structure and function. Glial scarring is the main obstacle to nerve regeneration [26, 27] and warrants further research. Acquiring suitable graft cells is the critical element that could ensure treatment efficacy. Currently, more and more researches are focused on improving the bioprocessing method to produce appropriate cells for tissue engineering.

Our previous findings have demonstrated that NSCs tend to differentiate into neurons and that glial differentiation is inhibited during spaceflight. Considering that microgravity is a key factor in space, we have here investigated the effects of microgravity on NSC function. Studies have demonstrated that microgravity contributes to providing cells for cell therapy [28, 29]. Most notably, emerging evidence indicates that microgravity affects not only cell differentiation but also the therapeutic effect of transplanted cells. The application of simulated microgravity for stem cell-based therapy in the treatment of CNS diseases has been reported [30]. The RCCS bioreactor was designed by the US National Aeronautics and Space Administration (NASA) and mimics the microgravity in space [31]. It has been shown that the RCCS system is superior to static culture conditions in some cases [32]. In this study, we evaluated whether the RCCS system can be used as a tool to provide suitable NSCs for future cellular therapies in neurology.

Compared with traditional 2D culturing on flat dish surfaces, scaffolds provide a 3D environment for cells to stretch out in all directions. Here, we used a collagen sponge scaffold. Our data indicate that NSCs grow and attach well on the scaffold, suggesting that this scaffold may be the optimal cell carrier for nerve tissue engineering and may provide a valuable platform to produce seeding cells. Furthermore, the NSCs attached to the collagen sponge scaffold are convenient to graft during cell transplantation for cell therapy. RNA sequencing and RT-PCR analysis revealed elevated NTRK3 expression under microgravity conditions in the RCCS system. It had been reported that NTRK3 overexpression may increase the capacity for neuronal differentiation [33]. It is well known that the neurotrophin NT-3 plays an important role in regulating neuronal differentiation and nerve regeneration. Nevertheless, neural precursor cells do not express sufficient levels of Trk receptors; this limits the positive effect of NT-3 on nerve regeneration. Therefore, establishing a method to cultivate NSCs in RCCS bioreactors is a promising strategy for tissue engineering. Compared to gene modification, this method is simpler, faster, and more easily adaptable to obtaining NSCs with high NTRK3 expression.

To enable the over-expressed NTRK3 to exert the biggest impact, a sufficient concentration of NT-3 in the differentiation medium is crucial. Our findings demonstrated that NTRK3-overexpressing cells promote migration and early neuronal differentiation and also suppress astrocytic differentiation. Furthermore, we demonstrated that NTRK3 is the target gene of let-7i-5p. A previous study demonstrated that inhibiting let-7i-5p enhances neuroprotection and facilitates functional recovery following stroke [34]; however, the functions of let-7i-5p in NSCs remain unclear. By performing luciferase assay and Western blot, we validated a direct regulatory role between NTRK3 and let-7i-5p that was predicted by our bioinformatics analysis. We also demonstrated that the positive effect of let-7i-5p-inhibitor on neuronal differentiation and migration in NT-3-containing differentiation medium can be reversed by adding NTRK3 siRNA.

## Conclusions

In summary, our results indicate that 3D cultured NSCs in RCCS bioreactors exhibit better neuronal differentiation and migratory ability than traditional static cultures. This is important for cell transplantation, as excellent neuronal differentiation and migratory ability increase treatment efficacy. Our initial findings suggest that employing a RCCS bioreactor in combination with NT-3 is a useful strategy to provide effective NSCs for stem cell therapy. The combination of the RCCS bioreactor and NT-3-containing medium may provide an attractive solution for therapeutic cell therapy demands in the future.

## Data Availability

The datasets supporting the conclusions of this article are included within the article.

## Abbreviations

RCCS: Rotary Cell Culture System
NTRK3: Neurotrophic receptor tyrosine kinase 3
NSCs: Neural stem cells
NT-3: Recombinant neurotrophin 3
3D: Three-dimensional
CNS: Central nervous system
2D: Two-dimensional
SD rats: Sprague-Dawley rats
GFP: Green fluorescent protein
SEM: Scanning electron microscope
FDA: Fluorescein diacetate
UTR: Untranslated region
Q-PCR: Quantitative real-time PCR
siRNAs: Small interfering RNAs
Tuj1: Neuronal class III -tubulin
Map 2: Microtubule-associated protein 2
GFAP: Glial fibrillary acidic protein
ANOVA: Analysis of variance
SCI: Spinal cord injury
NASA: US National Aeronautics and Space Administration
